# The impact of China's zero markup drug policy on drug costs for managing Parkinson's disease and its complications: an interrupted time series analysis

**DOI:** 10.3389/fpubh.2023.1159119

**Published:** 2023-05-09

**Authors:** Ruilin Wang, Xinya Li, Xinchun Gu, Qian Cai, Yayong Wang, Zhan-Miao Yi, Li-Chia Chen

**Affiliations:** ^1^Department of Pharmacy, Peking University Third Hospital, Beijing, China; ^2^Department of Pharmacy Administration and Clinical Pharmacy, School of Pharmaceutical Sciences, Peking University, Beijing, China; ^3^Institute for Drug Evaluation, Peking University Health Science Center, Beijing, China; ^4^Therapeutic Drug Monitoring and Clinical Toxicology Center, Peking University, Beijing, China; ^5^Division of Pharmacy and Optometry, Centre for Pharmacoepidemiology and Drug Safety, School of Health Sciences, Faculty of Biology, Medicine and Health, University of Manchester, Manchester Academic Health Science Centre, Manchester, United Kingdom

**Keywords:** interrupted time series, zero markup drug policy, Parkinson's disease, drug costs, disease burden study

## Abstract

**Background:**

In April 2009, the Chinese government launched Zero Markup Drug Policy (ZMDP) to adjust medical institutions' revenue and expenditure structures.

**Objective:**

This study evaluated the impact of implementing ZMDP (as an intervention) on the drug costs for managing Parkinson's disease (PD) and its complications from the healthcare providers' perspective.

**Methods:**

The drug costs for managing PD and its complications per outpatient visit or inpatient stay were estimated using electronic health data from a tertiary hospital in China from January 2016 to August 2018. An interrupted time series analysis was conducted to evaluate the immediate change following the intervention (step change, β_1_) and the change in slope, comparing post-intervention with the pre-intervention period (trend change, β_2_). Subgroup analyses were conducted in outpatients within the strata of age, patients with or without health insurance, and whether drugs were listed in the national Essential Medicine List (EML).

**Results:**

Overall, 18,158 outpatient visits and 366 inpatient stays were included. Outpatient (β_1_ = −201.7, 95%CI: −285.4, −117.9) and inpatient (β_1_ = −372.1, 95% CI: −643.6, −100.6) drug costs for managing PD significantly decreased when implementing ZMDP. However, for outpatients without health insurance, the trend change in drug costs for managing PD (β_2_ = 16.8, 95% CI: 8.0, 25.6) or PD complications (β_2_ = 12.6, 95% CI: 5.5, 19.7) significantly increased. Trend changes in outpatient drug costs for managing PD differed when stratifying drugs listed in EML (β_2_ = −1.4, 95% CI: −2.6, −0.2) or not (β_2_ = 6.3, 95%CI: 2.0, 10.7). Trend changes of outpatient drug costs for managing PD complications significantly increased in drugs listed in EML (β_2_ = 14.7, 95% CI 9.2, 20.3), patients without health insurance (β_2_ = 12.6, 95% CI 5.5, 19.7), and age under 65 (β_2_ = 24.3, 95% CI 17.3, 31.4).

**Conclusions:**

Drug costs for managing PD and its complications significantly decreased when implementing ZMDP. However, the trend in drug costs increased significantly in several subgroups, which may offset the decrease at the implementation.

## 1. Introduction

To mitigate the economic incentives of prescriptions, the Chinese government launched the Zero Markup Drug Policy (ZMDP) in 2017 to ban the markup on drug procurement in Health Care Institutions (HCIs). Since the 1980s, Chinese Public HCIs have been allowed to charge over 15% of the drug price as a service fee. This revenue led to supplier-induced demand (SID) for increasing drug expenditure in public hospitals (1, 2). In light of this, the ZMDP were implemented in public tertiary hospitals in Beijing in April 2017 as one of the various measures to reform the revenue and expenditure structures in public hospitals, such as increasing fees for labor-intensive services and reducing fees for diagnostic tests, which significantly impacted the composition of total direct medical costs (3–5).

Notably, to promote the availability and affordability of drugs, the National Essential Medicine Policy (NEMP) was launched in 2009, which may also impact drug costs (6). The purpose of essential medicines is to ensure that low- and middle-income countries have access to medicines for priority medical problems (7, 8). Therefore, the National Essential Medicines List (NEML) is considered to be the basis for public procurement or reimbursement to provide all citizens with equal access to basic health care with reasonable quality and financial risk protection. In addition, the government keeps expanding social health insurance coverage, but many cross-region encounters still could not be reimbursed due to the separate administration and operation nationally and locally (9, 10).

Parkinson's disease (PD) is a progressive neurodegenerative disease with an incidence rate of 1.7% in the older population in China. It requires long-term medication to manage the symptoms (11). The drug cost of managing PD and its complications is a heavy economic burden on healthcare systems and patients (12–14). Non-persistent medication use may impair disease control and incur complications of PD (e.g., depression, insomnia, and dementia), leading to increased disease burden and overall healthcare expenditures (15–17). Previous studies on antibiotics, anticancer, chronic obstructive pulmonary disease and other non-communicable diseases have found that implementing ZMDP reduced the drug cost and its weight in the direct medical cost (18–23). However, little is known about the impact of the ZMDP on the drug cost for patients with PD. Our previous research on healthcare costs for patients with PD found that direct medical and drug costs decreased gradually from 2016 to 2018. Nevertheless, the influencing factors need further exploration (24).

Theoretically, drug costs in Chinese HCIs are expected to decrease immediately after the implementation of the ZMDP in April 2017. However, the ZMDP's impact on the cost of drugs for managing PD and its complications may vary by the price elasticity of demand between drugs (25). Moreover, the impact of ZMDP on drug cost composition in different patient groups has not been comprehensively evaluated. The long-term impact of ZMDP on changes in drug costs and medication-taking behaviors is also unclear. Consequently, whether the reduction in drug cost benefits patients with PD in the long term remains unknown. Therefore, we conduct a longitudinal study to investigate the ZMDP's impact on the drug costs of PD and its complication from a medical Center with a neurological speciality, where movement disorders specialists formulate and adjust drug regimens of PD.

In addition, aging and other policy-related factors may influence drug costs for patients with PD (26). Previous studies have found that patients' age, patients with or without health insurance (27, 28), and whether the drug is listed in the Essential Medicines List (EML) (29–31) influenced Chinese physicians' prescribing behaviors (20, 27–29). The impact of these factors and the implementation of ZMDP on the drug cost in patients with PD is also unknown, and the policy effect on different patient groups might be various. Therefore, we also conducted subgroup analyses to explore the impact of implementing ZMDP on drug costs of patients in different age groups, with or without health insurance and whether drugs are listed in the national EML.

## 2. Method

### 2.1. Study design and data source

A quasi-experimental design was adopted to evaluate the impact of the ZMDP on drug costs using the electronic medical record (EMR) from January 1st, 2016, to August 15th, 2018, at Peking University Third Hospital (PUTH), a tertiary general medical center and teaching institution in Beijing, China. The PUTH EMR database contains individual patients' outpatient visits and inpatient admission datasets covering the patient ID, number of visits, visiting date (outpatient), admission and discharge date (inpatient), gender, age, type of medical insurance, diagnosis and costs of patients, and the name, quantity and price of drugs at PUTH.

Patients were included if they had a PD diagnosis in EMR identified using the International Classification of Diseases, Tenth Revision, Clinical Modification code (ICD-10 code: G20) and relevant disease terms (e.g., Parkinson). Patient characteristics and prescriptions of the study cohort were extracted for analysis. This study was nested within a protocol approved by the Institutional Ethics Board of Peking University Third Hospital (IRB00006761-M2018228) in 2018.

### 2.2. Outcome measures

In this study, the complications of PD included in our analysis were depression, insomnia, and dementia, which have the highest incidence (22). Each month, total drug costs for managing PD and its three complications were summed for outpatient and inpatient departments and divided by the number of outpatient visits, or inpatient stays to generate four series of the average drug cost. Costs were measured in the Chinese yuan (¥) after inflation adjustment using the medical care component of the Consumer Price Index (32, 33).

Due to the limited sample size of the inpatient stays, only the outpatient drug costs were stratified into subgroups to explore factors that might be associated with ZMDP's impact. The drug cost per outpatient visit was further stratified by patients' health insurance status (all sample drugs were in the National Reimbursement Drug List) and age (Age < 65, 65 ≤ age < 75 or age ≥ 75) for subgroup analysis. In the study, patients without health insurance mainly included self-pay patients and those whose health insurance did not cover health services in PUTH. Moreover, the drug cost for PD treatment per outpatient visit was further stratified into EML (levodopa/benserazide, amantadine and trihexyphenidyl) and non-EML drugs (selegiline, pramipexole, piribedil, carbidopa/levodopa and entacapone) according to the 2012 edition of the EML in China (34–36). Likewise, the drug cost for PD-related complications per outpatient visit was classified by drugs listed in EML (fluoxetine, paroxetine, mirtazapine, estazolam and zopiclone) and not listed in EML (sertraline, eszopiclone, zolpidem, donepezil, memantine and rivastigmine).

### 2.3. Statistical analysis

Interrupted time series (ITS) analysis was conducted to evaluate the effect of the ZMDP on the drug cost per outpatient visit or inpatient stay. The monthly time-series data from 2016 to 2018 were divided into the pre-intervention period (January 1st, 2016, to March 31th, 2017) and post-intervention (April 1st, 2017, to August 15th, 2018) periods by the implementation of ZMDP in April 2017, the following 3 months were tested for the possible policy lag effect. The interrupted time-series regression model is presented as the following equation. Step change (β_1_) and trend change (β_2_) were estimated and reported for each time series. The step change (β_1_) is an immediate change following the policy intervention. The trend change (β_2_) is a change in slope comparing the post-intervention with the pre-intervention period. The model was set up according to Schaffer et al. (37).


(1) $$\begin{array}{l} Y_{t}=\beta_{0}+\beta_{1}\times interruption+\beta_{2}\times time\;after\;interruption_{t}+e_{t} \end{array}$$

Dickey-Fuller and Durbin-Watson tests were used to test the stationarity and serial autocorrelation. If the non-stationarity or autocorrelation exists, then the autoregressive integrated moving average (ARIMA) model (38) was applied. Autoregressive (AR) refers to a model that uses the past values of a time series to predict its future values. Integrated (I) refers to the use of differencing to make the time series stationary, which means that its statistical properties, such as the mean and variance, remain constant over time. Moving Average (MA) is a model that uses past forecast errors to predict future values. ARIMA combines these three components (AR, I, and MA) to create a model that can handle non-stationary time series data. The parameters of an ARIMA model include the number of autoregressive terms (*p*), the degree of differencing (*d*), and the number of moving average terms (*q*).

As depicted in the following equation, in the ARIMA model, φ is the magnitude of the autocorrelation, θ is the value of the autocorrelation of the errors, *p* is the number of lags of the autoregressive model, *q* is the number of lags of the moving-average model, *d* is the degree of non-seasonal differencing, *L* is the lag operator, and ε_*t*_ is the error term (37, 39, 40).


(2) $$\begin{array}{l} \left(1-\sum_{i=1}^{p}\phi_{i}L^{i}\right){\left(1-L\right)}^{d}X_{t}=\left(1+\sum_{i=1}^{q}\theta_{i}L^{i}\right)\varepsilon_{t}\left(d\;\in \mathbb{Z},\;d>0\right) \end{array}$$

The autocorrelation function (ACF) and partial autocorrelation function (PACF) were used to determine the appropriate time series model (41). The goodness-of-fit between different models was assessed by the Ljung-Box test (42), the Akaike (AIC) and Schwarz or Bayesian (BIC) information criteria (43, 44).

The coefficient and coefficient and 95% confidence interval of the step change (β_1_) and trend change (β_2_) derived from the ARIMA model were summarized in tables. Besides, results from the linear regression (data before and after intervention), the counterfactual trend (predicting results in the absence of the intervention), and the ARIMA model (controlling for autocorrelation, seasonality, or non-stationarity) were presented in graphs. Moreover, based on the step and trend change parameter estimates, the absolute changes were calculated by the difference between the predicted pre-intervention trend of the outcomes and the estimated trend at the end of the study period. The absolute change assessed the relative changes as a relative proportion.

Furthermore, seven subgroup analyses were conducted on outpatients, including drugs listed in the EML, drugs not listed in the EML, patients with health insurance, patients without health insurance, patients < 65 years old (age < 65), patients between 65 and 75 years old (65 ≤ age < 75), and patients no < 75 years old (age ≥ 75).

All statistical analyses were performed with the software Microsoft Office Excel 2019 (Microsoft Corp.) and Stata version 15 (Stata Corp. LP) (45).

## 3. Results

**Figure 1:**
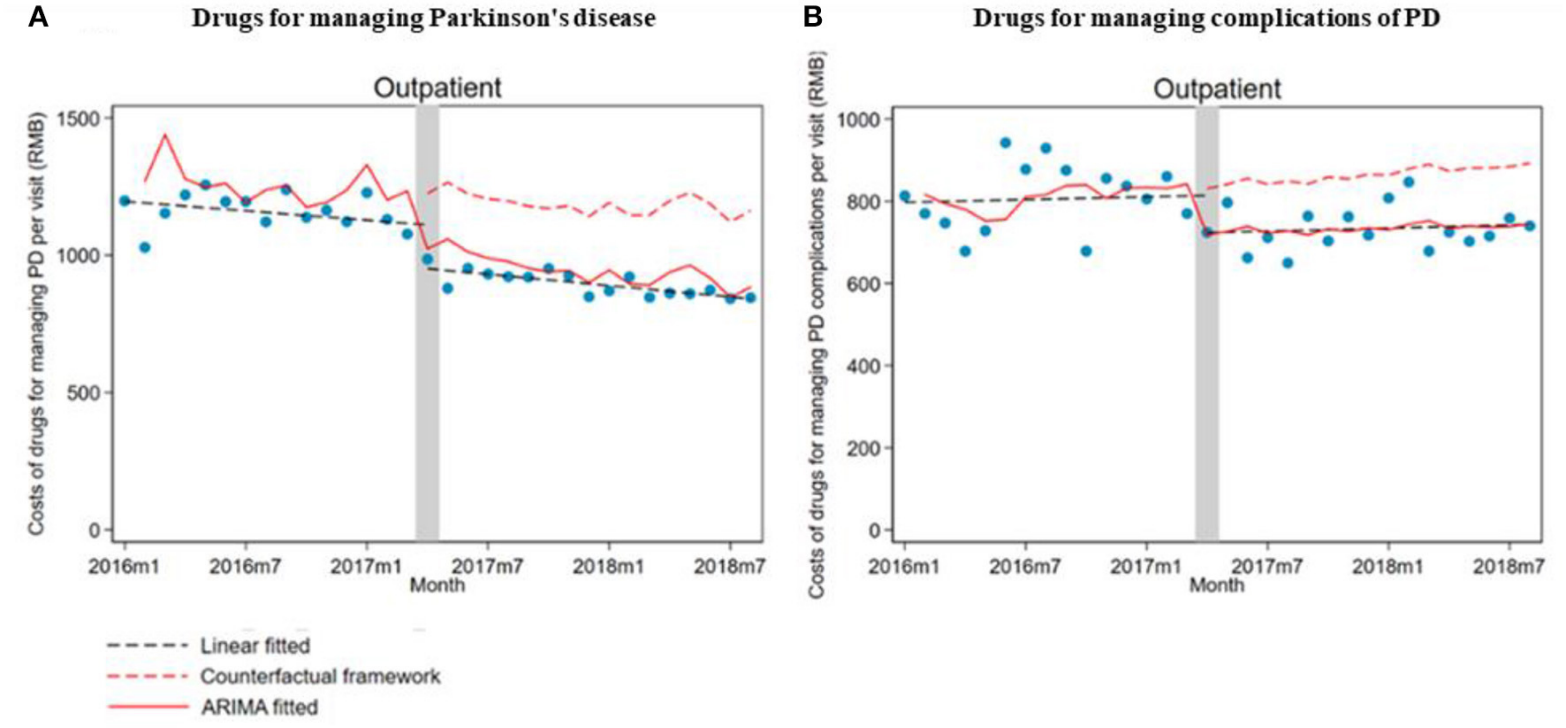
The impact of the Zero Mark-up Drug Policy on drug costs per outpatient visit for managing Parkinson's disease and its complications. **(A)** Drug costs for managing Parkinson's disease. **(B)** Drug costs for managing complications of Parkinson's disease.

In total, 18,158 outpatient visits and 366 inpatient stays were included in this study, with 2,640 outpatients and 330 inpatients. Outpatient visits and inpatient stays of medications for PD, and PD complications before and after the ZMDP were presented, respectively (Table 1). Due to the duplication between outpatient visits or inpatient stays with medications for PD and PD complications, there are differences between the summation results of numbers in Table 1 and the total number of outpatient visits or inpatient stays during the study period. There were 1,493 and 2,083 outpatients before and after the implementation of the ZMDP, respectively (Table 2). The drug costs for managing PD significantly decreased when the ZMDP was implemented (outpatients: β_1_ = −201.7; 95% CI: −285.4, −117.9; inpatients: β_1_ = −372.1; 95% CI: −643.6, −100.6). After implementing ZMDP, the drug costs for managing PD non-significantly changed (outpatient: β_2_ = −4.9; 95% CI: −14.6, 4.9, inpatient: β_2_ = 17.2; 95% CI: −10.3, 44.8) (Table 3; Figure 1; Supplementary Figure 1).

**Table 1:** Outpatient visits of subgroups and inpatient stays before and after the implementation of the Zero Markup Drug Policy.

**Subgroup**	**Before the zero markup drug policy**		**After the zero markup drug policy**	
	**Medications for PD**	**Medications for PD complications**	**Medications for PD**	**Medications for PD complications**
Total outpatient visits	7,253 (43.3%)	1,750 (42.2%)	9,487 (56.7%)	2,397 (57.8%)
Drugs listed in EML	4,683 (42.3%)	802 (47.3%)	6,393 (57.7%)	894 (52.7%)
Drugs not listed in EML	5,621 (44.0%)	1,084 (38.8%)	7,161 (56.0%)	1,713 (61.2%)
Patients with HI	4,500 (42.6%)	1,030 (41.0%)	6,069 (57.4%)	1,482 (59.0%)
Patients without HI	2,753 (44.6%)	720 (44.0%)	3,418 (55.4%)	915 (56.0%)
Age < 65	1,843 (43.2%)	240 (46.7%)	2,426 (56.8%)	274 (53.3%)
65 ≤ Age < 75	1,581 (41.7%)	293 (39.0%)	2,207 (58.3%)	458 (61.0%)
Age ≥ 75	3,829 (44.1%)	1,218 (42.2%)	4,854 (55.9%)	1,665 (57.8%)
Total inpatient stays	93 (48.9%)	43 (50.0%)	97 (51.1%)	43 (50.0%)

Outpatient visits or inpatient stays were counted as long as relevant medications were prescribed. PD, Parkinson's disease; EML, Essential Medicines List; HI, health insurance.

**Table 2:** Characteristics of outpatients before and after the implementation of the Zero Markup Drug Policy.

	**Before the zero markup drug policy**	**After the zero markup drug policy**
Male	804 (53.9%)	1,160 (55.7%)
Female	689 (46.1%)	923 (44.3%)
Age < 65	504 (33.8%)	684 (36.3%)
65 ≤ Age < 75	322 (21.6%)	278 (14.8%)
Age ≥ 75	667 (44.7%)	921 (48.9%)
Patients with HI	942 (63.1%)	1,321 (63.4%)
Patients without HI	551 (36.9%)	762 (36.6%)
Total	1,493	2,083

HI, health insurance.

**Table 3:** The impact of the Zero markup drug policy on the drug costs per outpatient visit or inpatient stay.

**Group**	**Step change (*β_1_*)**	**Trend change (*β_2_*)**	**Model**	**Absolute change**	**Relative change**
**Drug costs per outpatient visit for managing Parkinson's disease**					
Outpatient	−201.7 (-285.4, −117.9)^*^	−4.9 (-14.6, 4.9)	ARIMA (1, 1, 1)	−279.5 (-288.5, −270.6)_*_	−24.0% (-24.8%, −23.3%)^*^
Inpatient	−372.1 (-643.6, −100.6)^*^	17.2 (-10.3, 44.8)	ARIMA (0, 0, 0)	−96.1 (-363.3, 171.0)	−13.4% (-50.6%, 23.8%)
Drugs listed in EML	−33.7 (-44.6, −22.8)^*^	−1.4 (-2.6, −0.2)^*^	ARIMA (2, 1, 3)	−55.9 (-56.8, −55.0)^*^	−21.4% (-21.7%, −21.0%)^*^
Drugs not listed in EML	−181.7 (-222.7, −140.7)^*^	6.3 (2.0, 10.7)^*^	ARIMA (1, 1, 1)	−80.4 (-83.9, −77.0)^*^	−7.7% (-8.0%, −7.4%)^*^
Patients with HI	−159.5 (-390.4, 71.4)	0.9 (-20.2, 22.0)	ARIMA (1, 0, 3)	−355.4 (-427.5, −283.2)^*^	−31.9% (-38.4%, −25.4%)^*^
Patients without HI	−168.6 (-259.6, −77.5)^*^	16.8 (8.0, 25.6)^*^	ARIMA (3, 1, 3)	99.6 (92.4, 106.8)^*^	11.4% (10.6%, 12.2%)^*^
Age < 65	−241.4 (-385.6, −97.3)^*^	0.4 (-11.6, 12.5)	ARIMA (1, 0, 2)	−234.5 (-343.1, −126.0)^*^	−22.8% (-33.3%, −12.2%)^*^
65 ≤ Age < 75	−136.7 (-246.0, −27.3)^*^	−5.1 (-18.1, 8.0)	ARIMA (4, 0, 1)	−217.7 (-352.6, −82.8)^*^	−19.5% (-31.6%, −7.4%)^*^
Age ≥ 75	−155.0 (-217.3, −92.8)^*^	−1.6 (-8.8, 5.6)	ARIMA (1, 1, 3)	−181.2 (-185.6, −176.7)^*^	−17.6% (-18.0%, −17.2%)^*^
**Drug costs per outpatient visit for managing the complications of Parkinson's disease**					
Outpatient	−112.4 (-249.6, 24.8)	−2.2 (-16.2, 11.7)	ARIMA (1, 1, 1)	−148.3 (-160.8, −135.7)^*^	−16.6% (-18.0%, −15.2%)^*^
Inpatient	276.9 (-394.3, 948.1)	−13.5 (-80.9, 53.9)	ARIMA (0, 0, 0)	60.7 (-646.9, 768.2)	20.1% (-214.1%, 254.2%
Drugs listed in EML	41.2 (-15.7, 98.0)	14.7 (9.2, 20.3)^*^	ARIMA (1, 1, 1)	276.7 (274.3, 279.2)^*^	−142.0% (-140.7%, −143.2%)^*^
Drugs not listed in EML	−174.5 (-228.7, −120.3)^*^	−11.3 (-18.5, −4.1)^*^	ARIMA (1, 1, 1)	−144.8 (-161.9, −127.6)^*^	−12.9% (-14.5%, −11.4%)^*^
Patients with HI	−46.9 (-102., 8.3)	−5.0 (-12.4, 2.3)	ARIMA (2, 0, 2)	−127.5 (-201.5, −53.6)^*^	−15.1% (-23.9%, −6.4%)^*^
Patients without HI	−186.9 (-243.9, −130.0)^*^	12.6 (5.5, 19.7)^*^	ARIMA (3, 0, 3)	14.3 (-53.2, 81.7)	1.9% (-6.9%, 10.7%)
Age < 65	−142.7 (-231.8, −53.7)^*^	24.3 (17.3, 31.4)^*^	ARIMA (5, 0, 0)	246.4 (179.7, 313.1)^*^	54.0% (39.4%, 68.7%)^*^
65 ≤ Age < 75	−184.8 (-285.0, −84.6)^*^	7.2 (-4.2, 18.5)	ARIMA (1, 0, 1)	−70.1 (-174.2, 34.1)	−14.0% (-34.7%, 6.8%)
Age ≥ 75	−97.6 (-155.2, −40.0)^*^	0.6 (-6.1, 7.3)	ARIMA (1, 0, 1)	−88.5 (-155.5, −21.4)^*^	−9.2% (-16.2%, −2.2%)^*^

The results were presented in the coefficient and 95% confidence interval.

* P < 0.05; ARIMA, autoregressive integrated moving average; The parameters of an ARIMA model include the number of autoregressive terms (p), the degree of differencing (d), and the number of moving average terms (q). EML, essential medicine list; HI, health insurance; PD, Parkinson's disease.

**Figure 2:**
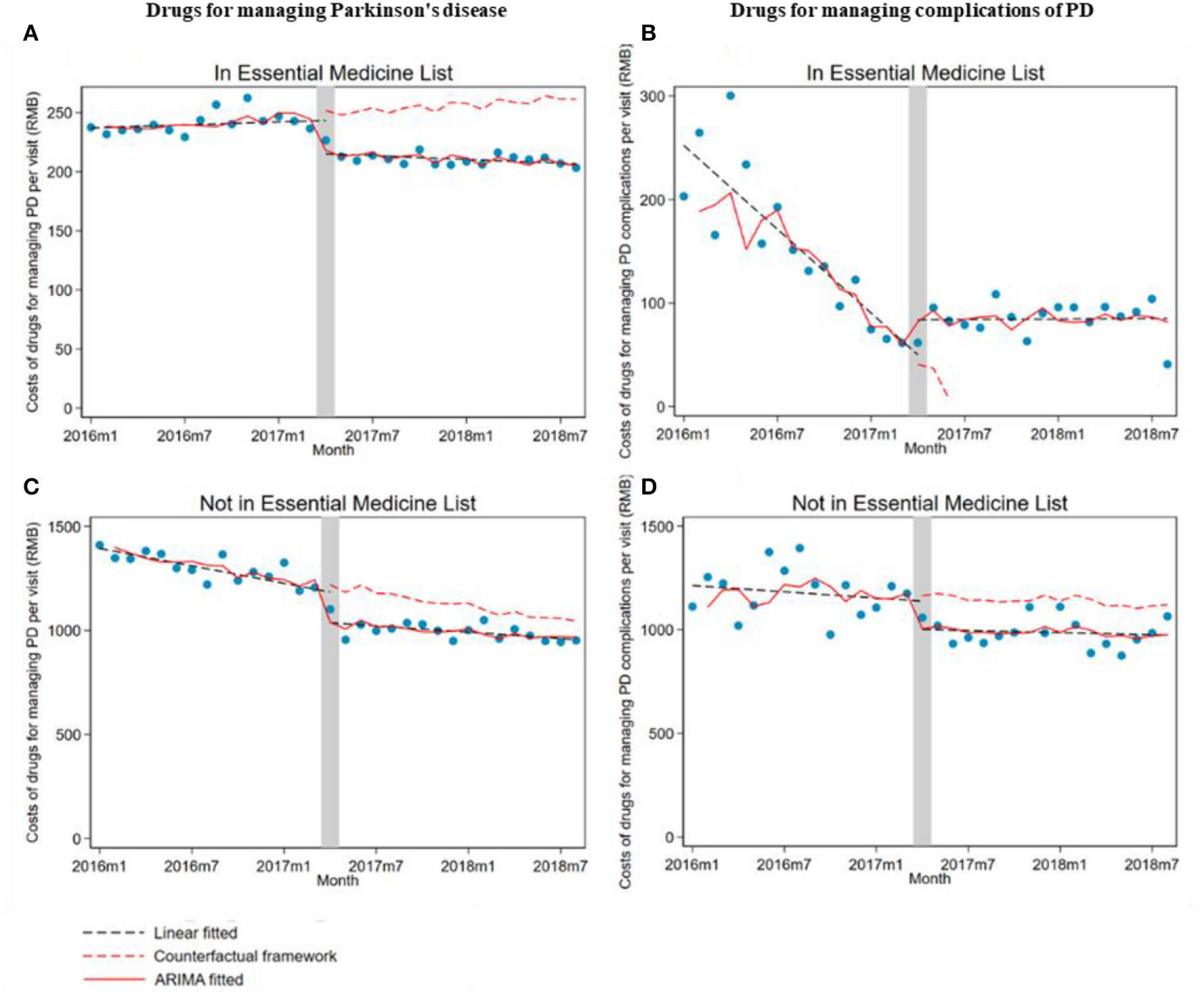
The impact of the Zero Mark-up Drug Policy on drug costs per outpatient visit of drugs, whether listed in EML for managing Parkinson's disease and its complications. **(A)** Drug listed in EML for managing Parkinson's disease. **(B)** Drug listed in EML for managing complications of Parkinson's disease. **(C)** Drugs not listed in EML for managing Parkinson's disease. **(D)** Drugs not listed in EML for managing complications of Parkinson's disease. EML, essential medicine list.

When stratifying by drug subgroups, the monthly drug cost per outpatient visit significantly decreased at implementing the ZMDP regardless of whether drugs are listed in EML (listed, β_1_ = −33.7; 95% CI: −44.6, −22.8; not listed, β_1_ = −181.7; 95% CI: −222.7, −140.7). Moreover, for drugs listed in EML for managing PD, the monthly trend significantly decreased (β_2_ = −1.4; 95% CI: −2.6, −0.2) comparing post-intervention with the pre-intervention period. However, the trend of drug costs significantly increased in post-intervention compared with the pre-intervention period for drugs listed in EML for managing PD complications (β_2_ = 14.7; 95% CI: 9.2, 20.3) and not listed in EML for managing PD (β_2_ = 6.3; 95% CI: 2.0, 10.7) (Table 3; Figure 2).

**Figure 3:**
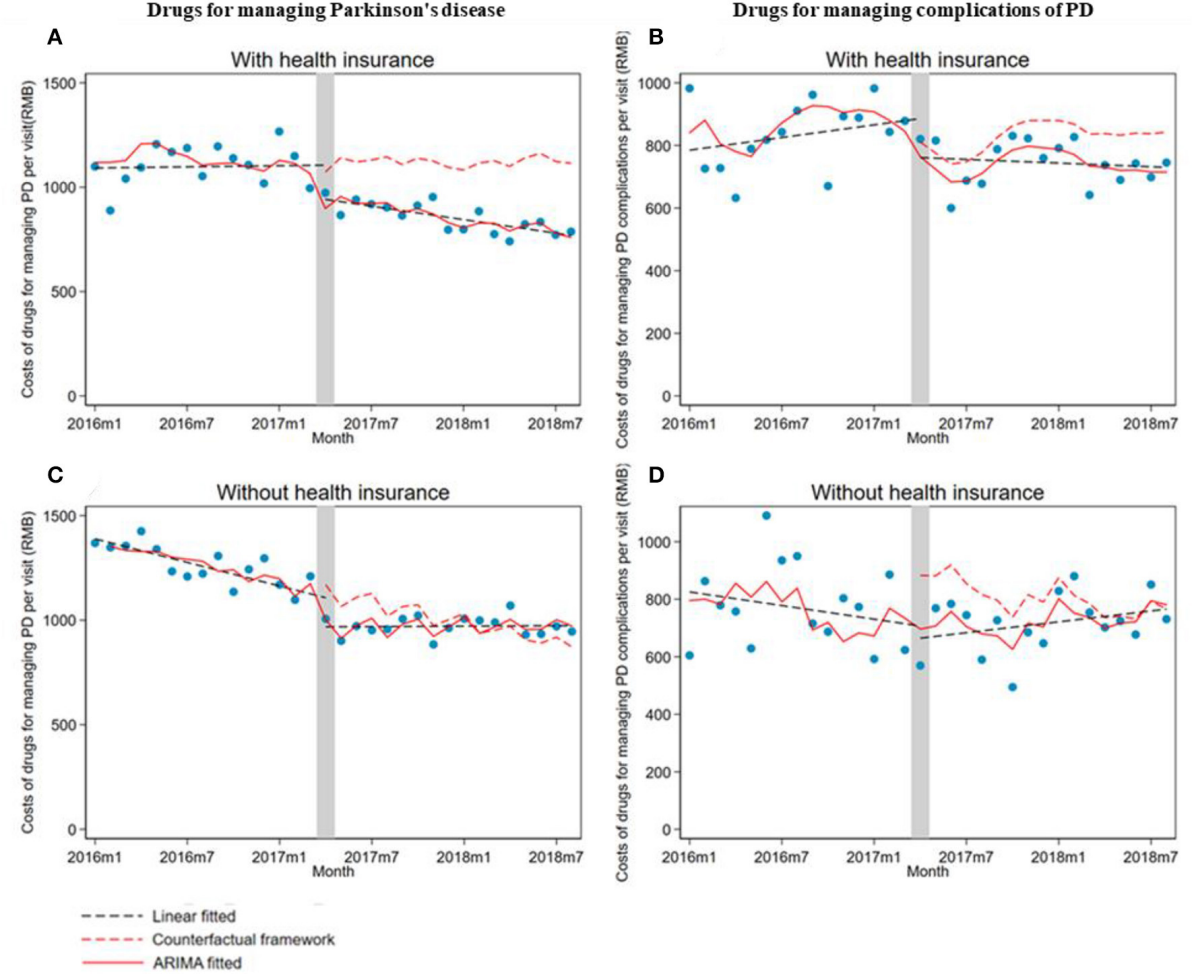
The impact of the Zero Mark-up Drug Policy on drug costs per outpatient visit of patients with or without health insurance for managing Parkinson's disease and its complications. **(A)** Drugs for managing Parkinson's disease in patients with health insurance. **(B)** Drugs for managing complications of Parkinson's disease in patients with health insurance. **(C)** Drugs for managing Parkinson's disease in patients without health insurance. **(D)** Drugs for managing complications of Parkinson's disease in patients without health insurance. HI, health insurance.

Monthly drug costs per visit for managing PD significantly (β_1_ = −168.6; 95% CI: −259.6, −77.5) and PD complications (β_1_ = −186.9; 95% CI: −243.9, −130) decreased in outpatients without health insurance. However, the trend changes in drug costs for managing PD (β_2_ = 16.8; 95% CI: 8.0, 25.6) and PD complications (β_2_ = 12.6; 95% CI: 5.5 to 19.7) increased in outpatients without health insurance compared with the pre-intervention period (Table 3; Figure 3).

**Figure 4:**
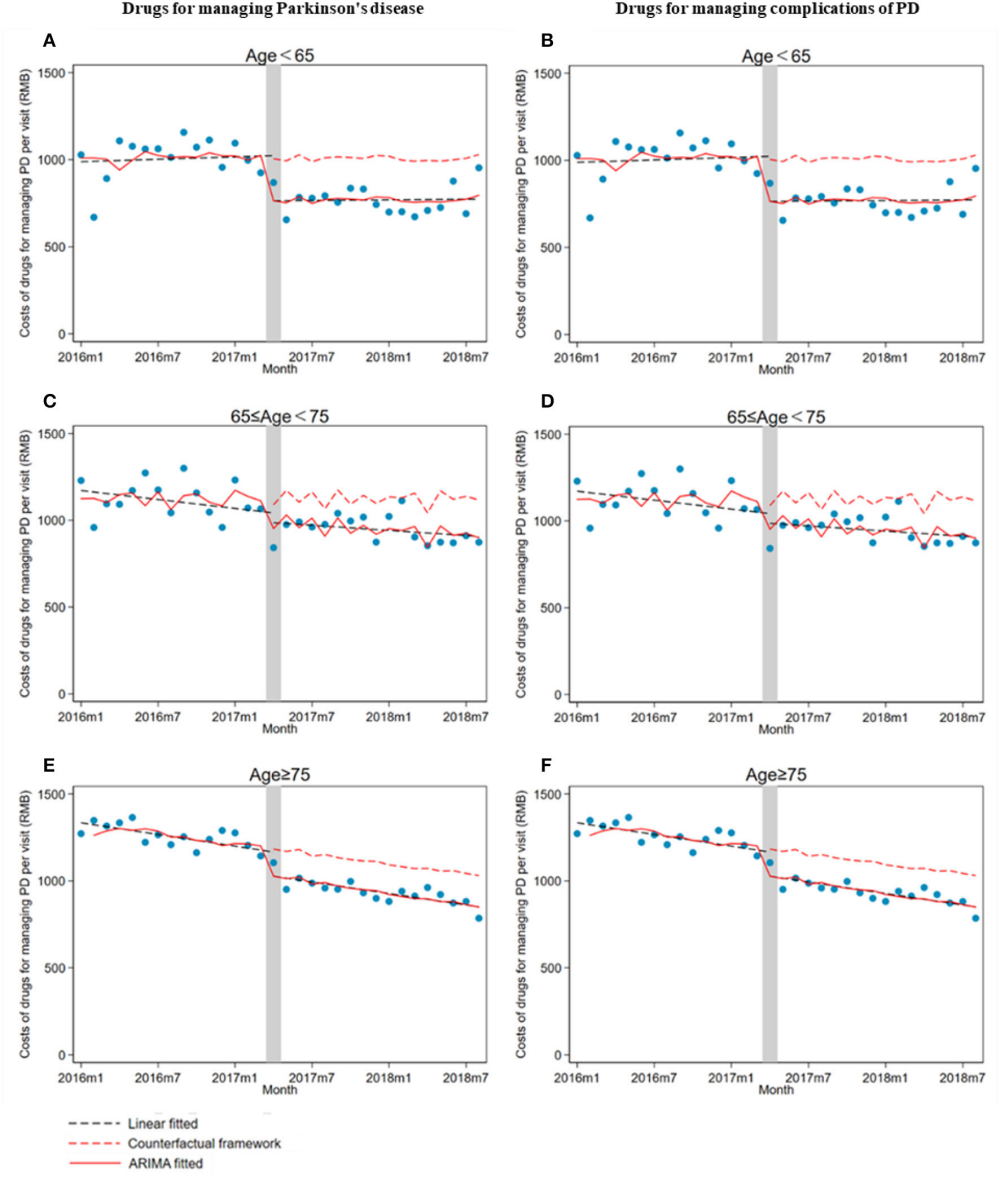
The impact of the Zero Mark-up Drug Policy on drug costs per outpatient visit for managing Parkinson's disease and its complications by age groups. **(A)** Drugs for managing Parkinson's disease in patients less than 65 years old. **(B)** Drugs for managing complications of Parkinson's disease in patients less than 65 years old. **(C)** Drugs for managing Parkinson's disease in patients greater than or equal to 65 years old and less than 75 years. **(D)** Drugs for managing complications of Parkinson's disease in patients greater than or equal to 65 years old and less than 75 years old. **(E)** Drugs for managing Parkinson's disease in patients greater than or equal to 75 years old. **(F)** Drugs for managing complications of Parkinson's disease in patients greater than or equal to 75 years old.

There was a significant decrease in drug costs (β_1_) for all age groups of outpatients when the ZMDP was implemented. Moreover, compared with the pre-intervention period, a significantly increasing trend change in drug costs for managing PD complications in outpatients under 65 years old was observed (β_2_ = 24.3; 95% CI: 17.3, 31.4) (Table 3; Figure 4).

## 4. Discussion

This study found that drug costs for managing PD significantly decreased with implementing the ZMDP. The impact of the ZMDP on the cost of drugs for managing PD or PD complications is in line with other studies, which demonstrated the universal and significant effects of policy on reducing the step and trend changes after policy implementation (20, 21). For example, Wang et al. (23) reported the implementation of the ZMDP was associated with a steep decline in drug costs for outpatients in 24 tertiary hospitals in Shanghai. However, in our study, compared with the pre-intervention period, the trend changes significantly increased in several subgroups when stratifying the cost of drugs for managing PD (drugs not listed in EML and patients without health insurance) or PD complications (drugs listed in EML, patients without health insurance and under 65 years old). The increase in trend change may likely offset the decrease in step-change compared with the pre-intervention period (46).

For those listed in EML, the trend change of drug costs per visit for managing PD significantly decreased after the ZMDP implementation. In contrast, opposite trend changes were shown for those not listed in EML, which may reflect the impact of China's national essential medicine list enabling patients to access appropriate, available, affordable, and quality essential medicines, especially in PD management. The World Health Organization Essential Medicines List (WHO-EML) was established to strengthen access to drugs of utmost importance, fundamental, indispensable and necessary for the health and needs of the population (47). In 2009, the Chinese government released the National Essential Medicines List, updated in 2012 and 2018. Besides, measures were implemented to strengthen the accessibility of these drugs, such as monitoring drug utilization, establishing the drug shortage list, and taking corresponding measures to ensure the supply of medicines (34–36). All these measures promote the use of essential medicines considered well-affordable and high-quality, which might contribute to the decrease in trend change compared with the pre-intervention period (48, 49).

Drug costs for PD and its complications in patients without health insurance significantly decreased in the step while increasing significantly in the trend compared with the pre-intervention period. This may be related to the change in physicians' prescribing behaviors to a certain extent (50–52). Since the drug cost decreased after the policy implementation, physicians might gradually prescribe more medication for managing PD and its complications because those medicines became more affordable after the price decreased. Once the decrease in drug costs is offset by the long-term upward trend for those without health insurance, the financial burden might be even heavier compared to the pre-international period (53). Therefore, policymakers should continue to expand health insurance coverage for patients and break through the regional barriers to health insurance coverage. Besides, for those subgroups with an increasing trend of outpatient drug costs, other policies are still needed to reduce the financial burden on PD patients, such as promoting the centralized purchasing policy and using generics (27).

It was found that while the cost of drugs for managing PD decreased, there was a long-term upward trend in the cost of drugs for managing PD among patients under 65 years old compared with the pre-intervention period. Consequently, the step decrease might be offset by the long-term upward trend, resulting in an increased financial burden to younger patients with PD. Patients with early-onset PD, usually under 65, have fewer complications than later-onset PD (54). Also, patients in different age groups vary in treatment preferences and medication compliance (55, 56). It is still unclear whether the increased drug costs for managing PD complications were due to the nature of disease progression or the irrational over-prescription motived by the reduced unit cost of drugs after the ZMDP. Further research is needed to evaluate the optimal medicine use associated with healthcare policies.

Furthermore, physicians' prescribing behaviors could be further optimized by actively promoting pharmacist services (57). After the implementation of ZMDP, the prices of some drugs, such as pramipexole, entacapone, and selegiline, decreased significantly, which might lead to more prescriptions for these drugs. In this process, clinical pharmacists specializing in neurology provided suggestions for drug therapy regimens, which reduced potential events of irrational drug use to some extent. Moreover, to promote the rational use of drugs, it is recommended to educate physicians on the value of relevant policies (28).

To the best of our knowledge, the study is the first to evaluate the effect of the ZMDP on drug costs for managing PD and its complications. We used the ARIMA model, a more flexible and powerful tool for modeling time-series data than simple linear regression, especially when the data exhibit autocorrelation, seasonality, or non-stationarity. The graphical presentation comparing results from linear regression, counterfactual frameworks and the ARIMA model gave a better insight into the policy's impact. The findings indicated the policy's effects varied in drugs for therapeutic and complication management and patients in different subgroups of patient age and health insurance. The subgroup analysis is valuable for investigating whether the policy disadvantaged vulnerable subgroups. Moreover, these insights can help policy decision-makers to adjust the policy further to ensure equitable access to health services. Besides, this study was conducted at a representative tertiary medical center in Beijing with a well-reputed neurological department. In this setting, neurologists' prescribing practices largely follow the clinical guideline. Therefore, this study's results represent the standard practice in medical centers with neurological specialities in China.

There are several worthy noting limitations to this study. Firstly, the single-center retrospective data may not fully represent the policy implementation's impact. The tertiary hospital could not represent all HCIs, especially the primary healthcare facilities. Therefore, further study on other medical settings is needed. Secondly, the retrospective data collected from EMR may introduce selection bias.

Nevertheless, in this study, patients were included according to the clinical diagnosis rather than prescribed drugs, thus ensuring drugs were likely prescribed for the intended purposes of interest to this study. Thirdly, changes in direct medical costs besides drug costs were not assessed due to the limited access to information. Although the final month (August 2018) did not include a whole month of data, we used the cost per visit as our outcome measure. Hence the impact is considered to be minimal. Finally, the impact of policy on appropriate drug use has not been thoroughly investigated. Further research is needed to investigate the optimal use of medicines by applying individual patient-level healthcare data.

## 5. Conclusion

We found that the ZMDP would reduce the financial burden on PD patients quickly to some extent. Still, in several subgroups, the significantly increasing trend in drug costs compared with the pre-intervention period might offset the decrease in drug costs at the implementing time. Drug prices may induce additional care needs for patients with chronic diseases, impacting the composition of patients' medical costs. Healthcare providers and policymakers must focus on the heterogeneity of policy's impacts and ensure care equity for different patient groups.

## Data availability statement

The raw data supporting the conclusions of this article will be made available by the authors, without undue reservation.

## Ethics statement

The study was approved by the Institutional Ethics Board of Peking University Third Hospital (IRB00006761-M2018228) in 2018.

## Author contributions

Z-MY and L-CC conceptualized the study and revised for the finalization of the manuscript. Z-MY, L-CC, RW, and XL designed the study. Z-MY acquired the funding and collected and assembled the data. RW, XL, XG, QC, and YW conducted data analysis and interpretation. RW and XL drafted the manuscript. All authors approved the final manuscript.
