# Supplementary material for: The impact of China's zero markup drug policy on drug costs for managing Parkinson's disease and its complications: an interrupted time series analysis
Source: Front Public Health. 2023 May 9;11:1159119. doi: 10.3389/fpubh.2023.1159119 (PMC10203530; doi:10.3389/fpubh.2023.1159119)
Supplement: Supplementary file 1 [file Table_1.docx]

# Supplementary Material

**The impact of China’s Zero Mark-up Drug Policy on drug costs for managing Parkinson’s disease and its complications: an interrupted time series analysis**

**Ruilin Wang ^1,2#^, Xinya Li ^1,2,3,4#^, Xinchun Gu ^5^, Qian Cai ^5^, Yayong Wang ^1^, Zhan-Miao Yi ^1,3,4^*^,^ Li-Chia Chen ^5^**

**Supplementary Figure 1.** **The impact of the Zero Mark-up Drug Policy on drug costs per inpatient stay**


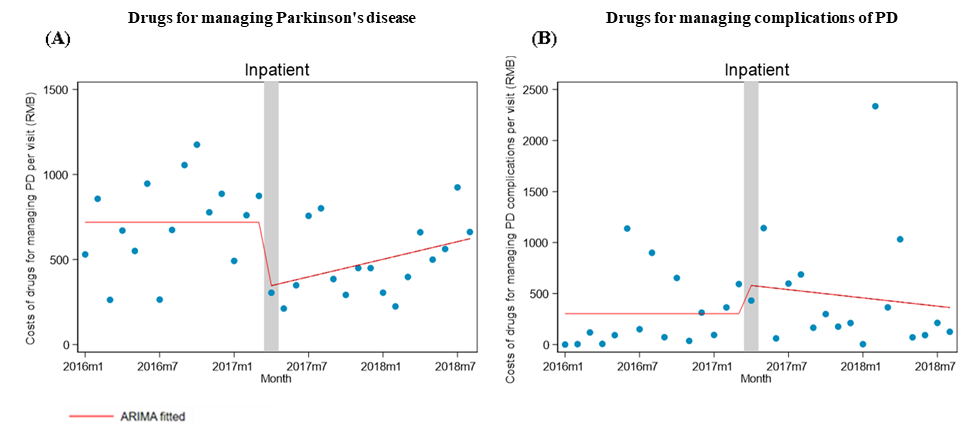


**Figure S1.** The impact of the Zero Mark-up Drug Policy on drug costs per inpatient stay for managing Parkinson’s disease and its complications.
